# Pre-Pregnancy Fast Food Consumption Is Associated with Gestational Diabetes Mellitus among Tehranian Women

**DOI:** 10.3390/nu9030216

**Published:** 2017-03-01

**Authors:** Minoor Lamyian, Somayeh Hosseinpour-Niazi, Parvin Mirmiran, Lida Moghaddam Banaem, Azita Goshtasebi, Fereidoun Azizi

**Affiliations:** 1 Department of Midwifery & Reproductive Health, Faculty of Medical Science, Tarbiat Modares University, 1411713114 Tehran, Iran; lamyianm@modares.ac.ir (M.L.); moghaddamb@modares.ac.ir (L.M.B.); 2 Nutrition and Endocrine Research Center, Research Institute for Endocrine Sciences, Shahid Beheshti University of Medical Sciences, 1985717413 Tehran, Iran; 3 Department of Clinical Nutrition and Dietetics, Faculty of Nutrition Sciences and Food Technology, National Nutrition and Food Technology Research Institute, Shahid Beheshti University of Medical Sciences, 1981619573 Tehran, Iran; 4 Health Metrics Research Center, Institute for Health Sciences Research, ACECR, 1981619573 Tehran, Iran; agoshtasebi@ihsr.ac.ir; 5 Endocrine Research Center, Research Institute for Endocrine Sciences, Shahid Beheshti University of Medical Sciences, 1985717413 Tehran, Iran; azizi@endocrine.ac.ir

**Keywords:** gestational diabetes mellitus, fast food consumption, French fries

## Abstract

The aim of this study was to evaluate the association between fast food consumption and gestational diabetes mellitus (GDM) among Tehranian women. This study was conducted over a 17-month period, on a random sample of pregnant women (*n* = 1026), aged 18–45 years, attending prenatal clinics in five hospitals affiliated with universities of medical sciences, located in different districts of Tehran, Iran. Dietary data were collected during gestational age ≤6 weeks, using a 168-item valid and reliable food frequency questionnaire. Consumption of total fast foods including hamburgers, sausages, bologna (beef), pizza and French fries was calculated. Between 24 and 28 weeks of gestation, all pregnant women underwent a scheduled 100 g 3 h oral glucose tolerance test. GDM was defined according to the American Diabetes Association definition. The mean age and pre-pregnancy body mass index BMI of participants were 26.7 ± 4.3 years and 25.4 ± 4.5 Kg/m^2^, respectively. A total of 71 women developed GDM. After adjustment for confounders, the OR (95% CI) for GDM for total fast food consumption was 2.12 (1.12–5.43) and for French fries it was 2.18 (1.05–4.70). No significant association was found between hamburgers, sausages, bologna (beef), pizza and GDM. Fast food consumption in women of reproductive age was found to have undesirable effects in the prevalence of GDM.

## 1. Introduction

Gestational diabetes mellitus (GDM), a relatively common pregnancy complication defined as glucose intolerance with onset or first diagnosed during pregnancy [1], has a strong association with the development of a number of unfavorable outcomes. These include complications for mothers, i.e., risk of impaired glucose tolerance (IGT) and type 2 diabetes in the years following pregnancy, and for offspring, i.e., obesity and developing IGT and diabetes in early adulthood [1]. The prevalence of GDM is increasing rapidly worldwide, making it a major public health challenge [1,2,3]. This global increase may be partly attributed to changes in dietary habits and nutritional transitions from traditional to Western-style fast food intake (high-energy and low nutrient density) which is steadily increasing and is a major factor in the development of metabolic disorders [4,5,6]. Although the undesirable effects of fast food consumption on obesity, insulin resistance and diabetes have been extensively studied [4,7,8,9], epidemiologic studies regarding the effects of fast food consumption in relation to GDM are scarce [10,11,12,13] and are mostly from Western populations that may not be generalizable to other populations because of cultural and social differences [14]. 

In Arab countries of the Middle East, during the past four decades, food consumption patterns have dramatically changed from traditional diets to westernized diets. In most of these countries there has been an increase in per capita energy (ranging from 10% in Sudan to 40% in Egypt) and fat (ranging from 13.6% in Sudan to 143.3% in Saudi Arabia) supplies, as well as high intakes of cholesterol and salt and low intakes of fiber [15]. Among Iranian adults, during the 1990s, a major characteristic of nutrition transition was a westernized diet containing junk and fast food which are high in fats, especially saturated and trans fatty acids [16]. In a previous study, mean fast food consumption among Iranian women was reported to be 122 g/week [17].

To our knowledge, there were no data available investigating the association of fast food consumption with GDM among Middle Eastern populations. Therefore, considering the nutritional transition in Middle Eastern populations, and the limited data available on the association between fast food consumption and GDM, we investigated the associations of pre-pregnancy consumption of fast food including French fries, hamburgers, bologna, and sausage, pizza and total fast food consumption and GDM among pregnant Tehranian women.

## 2. Materials and Methods

### 2.1. Subjects

This prospective study was conducted on 1045 women, attending prenatal clinics in five hospitals affiliated to universities of medical sciences (three prenatal clinics from each university randomly selected) in different districts, i.e., the south, west, east and north of Tehran, over a 17 month period, from August 2010 to January 2011. Inclusion criteria were being pregnant, age 18–45 years, singleton pregnancy, gestational age ≤6 weeks, gestations ≤2, and non-smokers. Participants were excluded if they had pre-existing diabetes (*n* = 12) or major chronic disease (*n* = 12), reported daily energy intakes outside the range of 800–4200 kcal/day (*n* = 7). The final study sample included 1026 participants. Written informed consent was obtained from all participants and the study protocol was approved by both the ethics committee of the faculty of Medical Sciences, Tarbiat Modares University, and the research council of the Research Institute for Endocrine Sciences (RIES), Shahid Beheshti University of Medical Sciences, Tehran, Iran.

### 2.2. Dietary Assessment

Mothers’ dietary intakes were assessed during gestational age ≤6 weeks, using a 168-item validated semi-quantitative food frequency questionnaire (FFQ) [18], designed to assess food intakes during the previous year. The FFQ consists of a list of foods with a standard serving size, commonly consumed by Iranians. Trained dieticians asked participants to designate their consumption frequency for each food item consumed during the previous year on daily, weekly, or monthly basis. The reported frequency for each food item was converted to a weekly intake; portion sizes of consumed food were then converted to grams using household measures [19]. Percentages of carbohydrate, fat and protein intake were calculated by multiplying the grams of consumption of each food by the content of carbohydrate, protein and fat. Composition values for carbohydrate, fat and protein were obtained from the US Department of Agriculture (USDA) FCT, because the Iranian Food Composition Table (FCT) is incomplete (limited to only raw materials and a few nutrients). In the present study, dietary variables including sausage, bologna (beef), hamburger, pizza, and French fries were considered as fast foods. Total consumption of fast food for each subject was calculated by summing up the weekly consumption of these foods. 

### 2.3. Diagnosis of GDM

Pregnant women underwent a scheduled 100 g 3 h oral glucose tolerance test (OGTT) between the 24th–28th weeks of gestation. Diagnosis of GDM was based on the criteria set by the American Diabetes Association. GDM was defined as the presence of at least two of the following four plasma glucose levels, measured while fasting and 1 h, 2 h and 3 h after the OGGT: (i) fasting ≥95 mg/dL; (ii) 1 h after ≥180 mg/dL; (iii) 2 h after ≥155 mg/dL; (iv) 3 h after ≥140 mg/dL [20].

Blood samples were drawn into vacutainer tubes in a sitting position, from all study participants. Serum fasting glucose concentration was assayed using an enzymatic colorimetric method with the glucose oxidase technique (Pars Azmoon Inc., Tehran, Iran). Inter- and intra-assay coefficients of variations were both 2.2% for serum glucose. 

### 2.4. Assessment of Other Variables

Weight was measured while the subjects were minimally clothed and not wearing shoes, using digital scales and was recorded to the nearest 100 g. Height was measured while subjects were standing without shoes, with their shoulders in a normal position, using a tape fixed to the wall and was recorded to the nearest 0.5 cm. Body mass index (BMI) was calculated as weight (kg) divided by square of height (m). Obesity was defined as a BMI 30–35 kg/m^2^. Physical activity was assessed using an oral questionnaire, including a list of common activities of daily life; the frequency and amount of time of activities spent per week over the past 12 months were documented [21]. Levels of physical activity were expressed as metabolic equivalent hours per week (METs h/week) [22]. Additional covariate information on age, family history of diabetes, history of GDM, exposure to smoking, and current use of medications was obtained using a questionnaire.

### 2.5. Statistical Analysis

Statistical analyses were performed using the Statistical Package for Social Science (version 15.0; SPSS Inc., Chicago, IL, USA). Characteristics of subjects at baseline are expressed as mean ± SD for continuous variables using one-way analysis of variance, and percentages for categorical variables using chi-square test across quartiles of fast food consumption. Energy-adjusted means for nutrients and food groups across quartiles of fast food consumption were determined using a general linear model analysis and total energy intake adjusted as a covariate. The trend of dietary variables was determined using linear regression by assigning the median value for each quartile of intake treated as a continuous variable. 

Participants were categorized according to quartiles of fast food consumption as follows: ≤42.6, 42.7–92.0, 92.1–175.0, and ≥175.1 g/week for quartiles one through four, respectively. Odds Ratio (OR) and their 95% confidence intervals for GDM were estimated using multivariable logistic regression models across quartiles of total fast food consumption and its components, adjusted for body mass index, physical activity levels, history of GDM, family history of diabetes, third gestational weight gain, age, level of education, total energy intake, total fiber, and cholesterol intake; in the multivariate model, the first quartile was considered as a reference. To determine *p* value for trends across quartile categories, we assigned the median intake of each quartile category to individuals’ variables in the logistic regression for GDM. *p* values < 0.05 were considered statistically significant. 

## 3. Results

Of 1026 study participants, 71 women had GDM, corresponding to 6.9% of pregnant women. The mean ± SD age and pre-pregnancy BMI of participants were 26.7 ± 4.3 years and 25.4 ± 4.5 kg/m^2^, respectively. Median intakes of hamburger, bologna (beef), pizza, sausage, and French fries and total fast food consumption were 3.7, 4.6, 13.1, 14.0, 29.6 and 92.0 g/week, respectively. Baseline characteristics of the participants across quartiles of fast food consumption are shown in Table 1. The proportion of history of GDM and BMI were higher in quartile 4, compared with those in quartile 1. No significant differences were found between the age, physical activity levels, family history of diabetes, gestational weight, gestational weight gain and exposure to smoking of participants in quartile 1 (the lowest quartile) and those in quartile 4 with fast food consumption. Energy-adjusted means for the nutrients and food groups of participants across quartiles of fast food consumption are shown in Table 2. Participants in the highest quartile of fast food consumption had significantly higher intakes of energy, carbohydrates, total fat, saturated fatty acids and cholesterol and lower intakes of fiber, fruit and whole grain than those in the lowest quartiles. 

The association between GDM and fast food consumption is shown in Table 3. Higher consumption of fast food consumption was positively associated with GDM risk, compared to women who consumed <42.6 g/week (reference group); the OR for those who consumed ≥175 g/week was 2.09 (95% CI 1.10–4.28; *p* for trend: 0.03), an association which remained significant after adjustment for confounding factors. Among types of fast foods, ORs (95% CIs) for GDM among participants with the highest, compared to the lowest, quartiles of French fries consumption were 2.32 (1.09–4.93); after adjustment for confounding factors, these associations, although attenuated, remained significant, with ORs of 2.18 (1.05–4.70). No association was found between hamburger, bologna, sausages or pizza consumption with GDM.

## 4. Discussion

In the current study, we found undesirable effects for pre-pregnancy fast food consumption on the incidence of GDM, independently of known risk factors for GDM, including BMI, physical activity and other potential confounding factors related to lifestyle. The odds of GDM increased over two-fold in participants who were in the highest quartile of fast food consumption. Among various fast foods, French fries played a more significant role in the occurrence of GDM. No statistically significant elevation in GDM was observed for other fast food items.

Our finding are in agreement with those epidemiologic studies which report undesirable effects of fast food consumption in relation to GDM [10,11,12,13]. A prospective study in the SUN project showed that pre-pregnancy higher consumption of fast food including high consumption of hamburgers, sausages, and pizza was associated with greater risk of incidence of GDM (OR, 1.86; 95% CI, 1.13–3.06) [10]. Another study also revealed that women in the highest quintile of the Western dietary pattern (higher consumption of red meat, processed meat, refined grain products, sweets, French fries and pizza) had a greater risk for GDM (OR, 1.63; 95% CI, 1.20–2.21) [11]. Furthermore, in another prospective study, during 10 years of follow-up, pre-pregnancy high consumption of sugar-sweetened cola (≥5 servings/week), compared with <1 serving per month, was positively associated with 22% greater GDM risk [12]. In addition, the prudent dietary pattern, defined as higher consumption of seafood, eggs, vegetables, fruit and lower consumption of soft drinks and French fries, was associated with lower GDM risk [13]. However, in a cross-sectional study, no association was found between the unhealthy retail food outlets such as fast food, pizza, bodegas, bakeries, convenience, candy/nut and meat stores in the neighborhood and gestational diabetes mellitus [23]. Moreover, in a prospective study, diet quality in the first trimester of pregnancy was not associated with risk of developing IGT or GDM [24], and maybe the relatively short duration of pregnancy may not allow time for the diet to affect the risk for GDM.

A major characteristic of fast food consumption is its high contents of fats, and saturated and trans-fats, [25]; in some studies, higher pre-pregnancy consumption of these components of fast foods was associated with an increased risk of gestational diabetes [26,27]. Our findings are in general agreement with those of these studies, since the components of fast food considered in our study (hamburgers, sausages, and pizza) mainly include a high fat content of up to 36% in hamburgers, and include a high content of trans fatty acids (23.6% to 30.6% of total fatty acids) and saturated fatty acids (21.5% to 38.4%) [25]. Data shows that high intakes of saturated fatty acids suppress activity of pancreatic *Mgat4a*-encoded GlcNAcT-IV glycosyltransferase [28], increase blood glucose and body weight, and induce placental oxidative stress and vascular dysregulation, conditions observed in GDM patients, which contribute to the pathogenesis of the disease [29]. 

Another potential explanation of fast foods is related to their energy density, which can disturb the regulation of appetite and result in a significant reduction in sensory-specific satiety, which is an important hedonic inhibitor of energy intake [30]. These disturbances lead to overweight, obesity, and insulin resistance, and therefore GDM [4]. In a prospective study, pre-pregnancy body mass index was a stronger predictor of GDM than diet quality during pregnancy [24]. A high weight gain rate during pregnancy may also be relevant to the pathophysiology of GDM [31,32]; the ‘fast food’ dietary pattern (characterized by higher consumption of sweets, soft drinks, hamburgers, pizza and other fast foods), in a dose-dependent manner, has also been associated with higher weight gain during pregnancy [33]. Moreover, dietary factors such as refined and high-glycemic-index carbohydrates are other threatening factors that may contribute to impaired insulin secretion and pancreatic β-cell function, and induced insulin resistance before pregnancy [34], and lead to GDM during pregnancy [12,35]. 

Likewise, high intakes of red and processed meat, which are fast foods, may be relevant in the pathophysiology of gestational diabetes [11,36]; in the Nurses’ Health Study II, for each increase of one serving/day of total red meat or processed red meat (beef, lamb, pork, hamburger, bacon, beef hot dogs and sausages, salami and bologna) before pregnancy, the GDM risk increased by 66% (95% CI 1.36%, 2.02%) and 47% (95% CI 0.98%, 2.20%), respectively [36]. In a prospective cohort study, the Western diet was positively associated with GDM risk, an association largely explained by intakes of red and processed meat products; the relative risk of GDM for each increment of one serving per day was 1.61 (95% CI 1.25–2.07) for red meat and 1.64 (95% CI 1.13–2.38) for processed meat [11]. High contents of saturated and trans fatty acids as well as nitrites (a preservative in processed meats), nitrosamines and advanced glycation end products in red and processed meat are reported to be associated with beta cell toxicity, insulin resistance and GDM risk [37,38].

Our study has several strengths; it investigated a general population of Tehranian women with a large sample size and the study’s population-based design enhances its generalizability. Moreover, we used a validated and reliable FFQ specifically developed for our population. In addition, for more accurate diagnosis of GDM, an OGTT was performed in all participants. As regards the limitations of the study, dietary intakes of participants were only assessed at the gestational age of ≤6 weeks in the previous year, and change in diet during pregnancy was not assessed. Therefore, we are unable to confirm that the association of pre-pregnancy diet and GDM is independent of diet during pregnancy. In addition, we could not determine causality between fast food consumption and the risk of GDM; however, our findings suggest that a pre-pregnancy diet was associated with susceptibility of women to GDM. Further research is required to investigate the contribution of change of diet during pregnancy to GDM risk.

## 5. Conclusions

In conclusion, findings from this study suggest that pre-pregnancy higher consumption of fast food is a risk factor for GDM, indicating that clinical and public health efforts, encouraging women of reproductive age to consume healthy diets, are essential to reduce GDM risk in a pregnancy.

## Figures and Tables

**Table 1:** Characteristics of participants by quartiles of fast food consumption ^a^.

Characteristics	Quartiles of Fast Food Consumption				*p* ^b^
	1	2	3	4	
Participants (*n*)	257	256	257	256	
Age (year)	27.1 ± 4.5	26.6 ± 4.1	26.5 ± 4.1	26.8 ± 4.5	0.47
Physical activity (MET h-week)	30.0 ± 2.9	31.2 ± 2.9	31.0 ± 2.9	29.1 ± 2.9	0.72
BMI (kg/m^2^)	25.2 ± 4.5	25.5 ± 4.8	25.7 ± 5.0	27.8 ± 6.2	0.01
Family history of diabetes, *n* (%)	35 (12.1)	34 (17.9)	39 (14.4)	38 (12.1)	0.91
First gestational weight (kg)	66.3 ± 12.5	65.3 ± 12.3	65.6 ± 12.0	66.0 ± 11.5	0.81
Second gestational weight (kg)	71.3 ± 12.2	70.4 ± 11.9	70.8 ± 11.9	70.8 ± 11.9	0.84
Third gestational weight (kg)	76.5 ± 12.2	75.5 ± 12.2	75.5 ± 11.8	75.4 ± 11.3	0.70
Weight gain in second trimester pregnancy (kg) ^c^	5.0 ± 4.9	5.0 ± 3.3	5.2 ± 3.2	4.8 ± 3.7	0.67
Weight gain in third trimester pregnancy (kg) ^d^	10.2 ± 4.9	10.1 ± 4.7	9.8 ± 4.8	9.4 ± 5.6	0.27
History of GDM, *n* (%)	7 (2.7)	3 (1.2)	1 (0.4)	1 (0.4)	0.05
Exposure to smoking (%)	30 (11.7)	38 (11.7)	38 (14.8)	29 (11.3)	0.60

BMI, Body mass index; GDM, gestational diabetes mellitus. ^a^ Mean ± SD for all these values, except for variables was determined; ^b^ ANOVA for continuous variables and chi-square test for categorical variables; ^c^ Weight gain in second trimester of pregnancy was calculated by subtracting the first gestational weight values from the second gestational weight values; ^d^ Weight gain in third trimester of pregnancy was calculated by subtracting the first gestational weight values from the third gestational weight values.

**Table 2:** Dietary intakes of participants by quartiles of fast food consumption.

Dietary Intakes	Quartiles of Total Fast Food Consumption				
	1	2	3	4	*p*
Hamburger (g/day)	2.29 ± 1.7	7.6 ± 1.7	13.2 ± 1.7	33.4 ± 1.7	<0.001
Sausages (g/day)	3.7 ± 6.1	12.8 ± 6.1	24.5 ± 6.1	74.7 ± 6.1	<0.001
Bologna (g/day)	1.6 ± 2.1	6.1 ± 2.1	14.3 ± 2.1	42.4 ± 2.1	<0.001
French fries (g/week)	10.6 ± 3.7	27.9 ± 3.7	42.3 ± 3.7	94.8 ± 3.7	<0.001
Pizza(g/day)	4.2 ± 3.5	12.3 ± 3.5	30.7 ± 3.5	95.7 ± 3.5	<0.001
Total energy (kcal/day)	2425 ± 51	2364 ± 51	2454 ± 51	2647 ± 51	0.001
Carbohydrate (% of total energy)	55.0 ± 0.8	56.3 ± 0.8	58.5 ± 0.8	57.2 ± 0.8	0.01
Protein (% of total energy)	13.3 ± 0.2	13.8 ± 0.2	13.5 ± 0.2	12.9 ± 0.2	0.03
Fat (% of total energy)	30.6 ± 0.5	31.2 ± 0.5	33.6 ± 0.5	34.0 ± 0.5	0.01
SFA (% of total energy)	10.7 ± 0.2	10.3 ± 0.2	11.2 ± 0.2	11.8 ± 0.2	0.01
MUFA (% of total energy)	10.6 ± 0.2	10.8 ± 0.2	10.9 ± 0.2	11.3 ± 0.2	0.11
PUFA (% of total energy)	7.0 ± 0.2	6.6 ± 0.2	6.5 ± 0.2	6.4 ± 0.2	0.08
Cholesterol (mg/day)	238 ± 9.8	229 ± 9.9	227 ± 9.8	263 ± 9.9	0.04
Magnesium (mg/day)	398 ± 14	406 ± 14	407 ± 14	417 ± 14	0.85
Fiber (mg/day)	26.6 ± 1.0	27.2 ± 1.1	21.3 ± 1.0	21.8 ± 1.1	<0.001
Vegetable (g/day)	318 ± 11	325 ± 11	347 ± 11	312 ± 11	0.13
Fruit (g/day)	339 ± 13	332 ± 13	297 ± 13	300 ± 13	0.05
Whole grain (g/day)	97.7 ± 8.5	94.4 ± 8.5	94.9 ± 8.5	80.9 ± 8.5	0.04
Meat, poultry and fish (g/day)	51.1 ± 2.9	45.9 ± 2.9	49.4 ± 2.9	55.2 ± 2.9	0.16
Legumes (g/day)	19.2 ± 1.6	19.1 ± 1.6	21.8 ± 1.6	23.4 ± 1.6	0.21
Nuts (g/day)	13.9 ± 1.2	12.8 ± 1.2	13.4 ± 1.2	15.2 ± 1.2	0.58
Dairy products (g/day)	10.6 ± 0.6	10.1 ± 0.6	11.3 ± 0.6	10.9 ± 0.6	0.51

SFA, saturated fatty acids; MUFA: mono-unsaturated fatty acids; PUFA, poly unsaturated fatty acids. Data are mean ± SEM (adjusted for energy intake).

**Table 3:** Multivariate adjusted odds ratio (95% CI) for GDM across quartiles of fast food items.

Fast Food Items	Quartiles				*p* for Trend
	1	2	3	4	
**Total fast food**					
Median intake (g/week)	22.5	67.2	121.1	284.0	
Range of intake (g/week)	≤42.6	42.7–92.0	92.1–175.0	≥175.1	
Model 1	1	1.34 (0.62–2.90)	1.63 (0.77– 3.43)	2.09 (1.10–4.28)	0.03
Model 2	1	1.30 (0.59–2.84)	1.53 (0.71–3.28)	2.12 (1.12–5.43)	0.03
**French fries**					
Median intake (g/week)	7.0	16.9	30.0	90.0	
Range of intake (g/week)	≤7.0	7.0–29.9	29.9–60.0	≥60.1	
Model 1	1	2.01 ( 0.93–4.31)	1.89 (0.88–4.02)	2.32 (1.09–4.93)	0.09
Model 2	1	1.88 (0.86–4.08)	1.80 (0.83–3.90)	2.18 (1.05–4.70)	0.12
**Hamburger**					
Median intake (g/week)	0.0	2.4	15.2	37.8	
Range of intake (g/week)	0.0	0.1–3.7	3.8–15.2	≥15.3	
Model 1	1	0.77 (0.40–1.50)	0.54 (0.28–1.10)	1.15 (0.59–2.25)	0.65
Model 2	1	0.66 (0.33–1.31)	0.52 (0.26–1.03)	1.13 (0.61–2.19)	0.41
**Bologna**					
Median intake (g/week)	0.0	4.6	9.3	35.0	
Range of intake (g/week)	≤0.7	0.8–4.6	4.7–18.6	≥18.7	
Model 1	1	0.76 (0.40–1.43)	0.38 (0.18–0.78)	0.81 (0.42–1.56)	0.08
Model 2	1	0.81 (0.41–1.57)	0.38 (0.18–0.80)	0.82 (0.42–1.61)	0.11
**Sausages**					
Median intake (g/week)	0.0	9.3	23.0	60.0	
Range of intake (g/week)	≤2.3	2.4–14.0	14.1–28.0	≥28.1	
Model 1	1	0.82 (0.41–1.65)	1.14 (0.53–2.43)	1.72 (0.89–3.32)	0.07
Model 2	1	0.84 (0.41–1.72)	1.14 (0.52–2.49)	1.70 (0.85–3.29)	0.07
**Pizza**					
Median intake (g/week)	0.0	12.0	26.2	105	
Range of intake (g/week)	≤2.2	2.3–13.1	13.2–39.4	≥39.5	
Model 1	1	0.62 (0.32–1.23)	1.29 (0.6–2.66)	1.24 (0.65–2.38)	0.17
Model 2	1	0.69 (0.35–1.37)	1.27 (0.61–2.64)	1.32 (0.68–2.57)	0.16

^a^ The median intake of each quartile category was assigned and then these quartile median variables were included as a continuous variable in logistic regression. Model 1 was crude. Model 2 was adjusted for body mass index, history of GDM, family history of diabetes, third gestational weight gain, age, level of education, total energy intake, total fiber, and cholesterol intake.
